# Case report: 177Lu DOTA-TATE: a new scheme for the treatment of prostate neuroendocrine cancer

**DOI:** 10.3389/fonc.2023.1289272

**Published:** 2023-12-11

**Authors:** Xin-yuan Yu, Yu-qin Zhu, Xin Liu, Rong Tian, Jun-jie Chen, Guo-qing Liu, Dong-yu Yang, Xue-ping Zhang, Bao Li, Hong-jun Zhao, Xiao Li

**Affiliations:** ^1^ Department of Urology Surgery, The First Affiliated Hospital of Weifang Medical University, Weifang, Shandong, China; ^2^ Department of Intensive Care, Affiliated Hospital of Weifang Medical University, Weifang, Shandong, China

**Keywords:** prostatic cancer, prostate small cell carcinoma, radionuclide therapy, 177Lu-DOTA-TATE, review of the literature

## Abstract

**Background:**

Most instances of small cell carcinoma originate from the lungs, while the gastrointestinal tract serves as a secondary site. Only a minuscule proportion of cases manifest within the urogenital system. Prostate small cell carcinoma (SCCP) represents an exceedingly uncommon pathological subtype within the realm of prostate cancer, displaying significant rarity in clinical settings. This scarcity has resulted in a paucity of adequate foundational and clinical research for SCCP treatment. While investigations have unveiled a certain therapeutic efficacy of radiotherapy and chemotherapy for SCCP, clinical practice has revealed suboptimal treatment outcomes. We hereby present a case report detailing the utilization of 177Lu-DOTA-TATE in the treatment of SCCP, aiming to investigate the therapeutic efficacy of 177Lu-DOTA-TATE for SCCP.

**Case presentation:**

A male patient in his 80s presented with elevated prostate-specific antigen (PSA) levels and underwent a biopsy that revealed prostate adenocarcinoma. The patient received CAB (bicalutamide + goserelin) therapy. One year later, disease progression was detected, and a second biopsy confirmed the presence of prostate small cell carcinoma. Following the diagnosis of prostate small cell carcinoma, the patient underwent two cycles of 177Lu-DOTA-TATE treatment. Subsequent to the treatment, the original lesions showed shrinkage, metastatic lesions disappeared, and there was significant improvement, approaching complete remission.

**Conclusion:**

SCCP exhibits a high degree of malignancy and aggressive invasiveness, currently lacking effective therapeutic modalities. The treatment course of this patient serves as compelling evidence for the efficacy of 177Lu-DOTA-TATE in managing SCCP, thereby opening new avenues for future SCCP treatments.

## Introduction

Neuroendocrine carcinoma of the genitourinary tract is an exceptionally rare malignancy, ranking as the third most prevalent extrapulmonary site after the gastrointestinal and pancreatic regions. SCCP, characterized by high invasiveness, is associated with poor patient prognosis. 177Lu-DOTA-TATE, a somatostatin analog labeled with ^177Lu, finds widespread application in the treatment of gastrointestinal neuroendocrine tumors. Herein, we systematically review the literature pertaining to SCCP, summarizing its clinical and pathological features, current treatment practices, prognosis, and the therapeutic outcomes following the administration of 177Lu-DOTA-TATE.

## Case presentation

**Figure 1:**
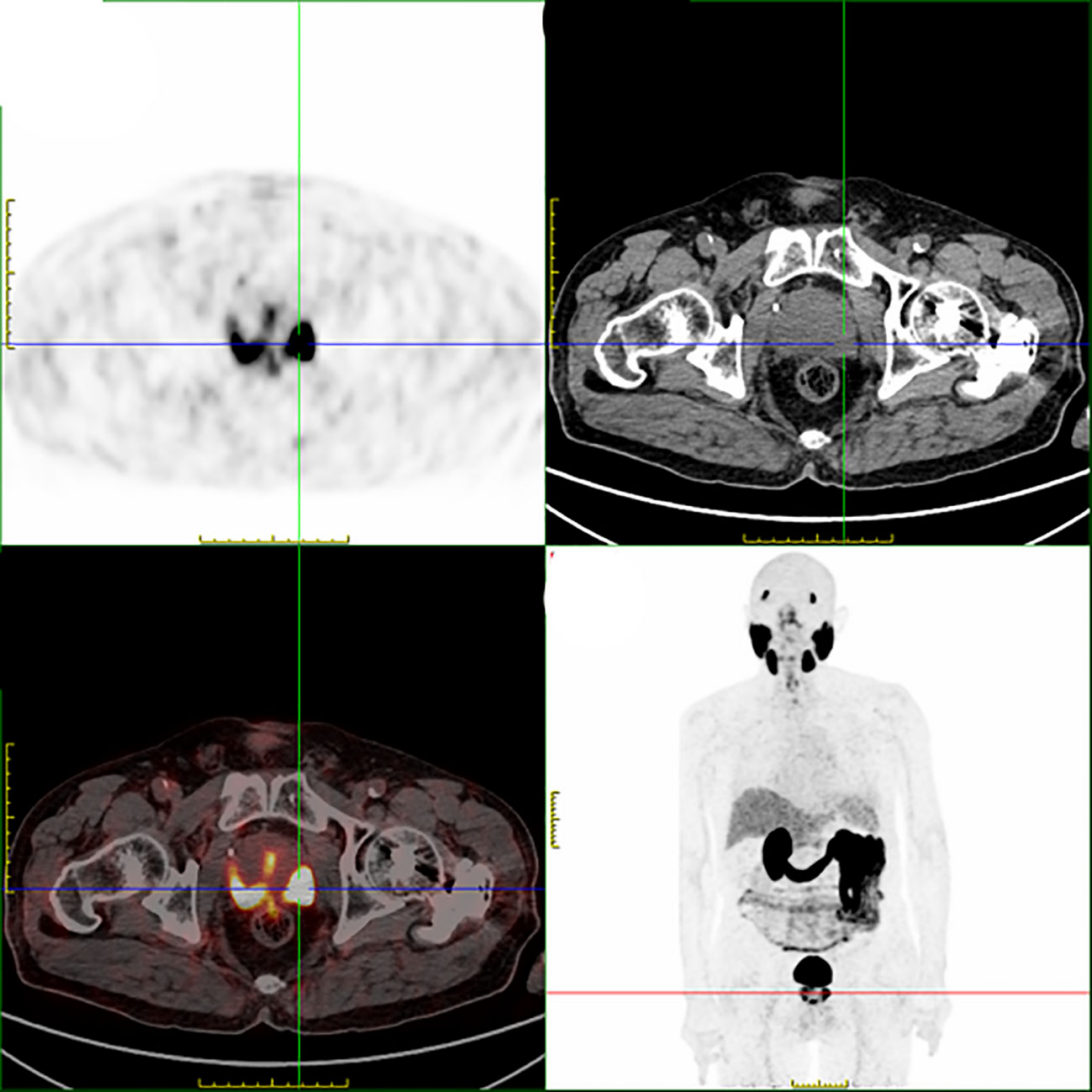
Enlarged prostate with multiple foci of high PSMA expression in the prostate, considering malignant lesions.

**Figure 2:**
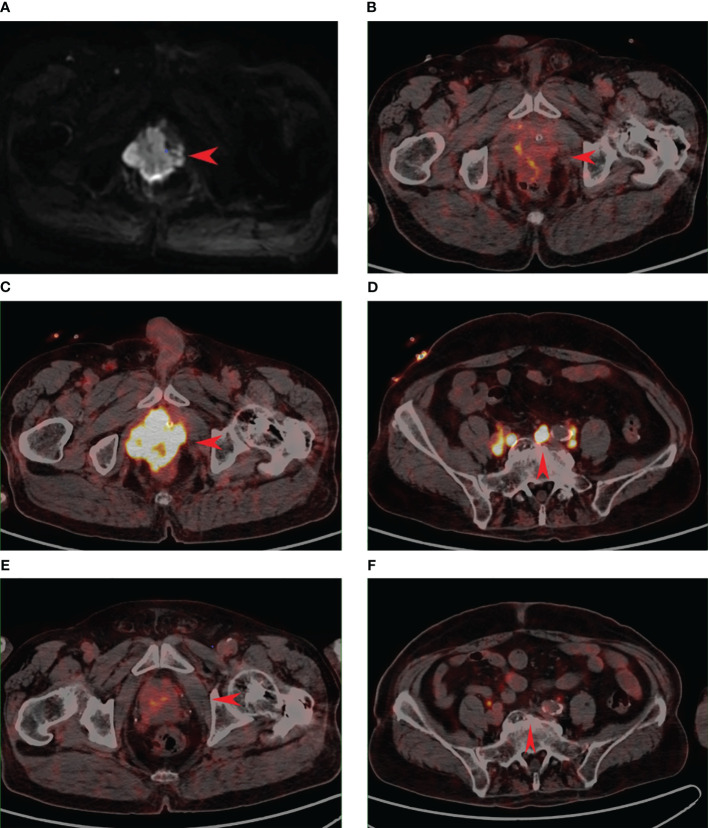
A.2022.5 Prostate MR scan: review after prostate cancer treatment to consider disease progression B.2022.5 PSMA PET suggests an irregularly enlarged prostate with heterogeneous density, uneven PSMA radioactivity distribution only slightly increased, SUVmax=4.8. **(B)** is inconsistent with the imaging performance of the lesion in **(A)**. C.2022.5 18F-OC PET suggests foci of high octreotide expression in the prostate, SUVmax=12.3. **(C)** is consistent with the imaging presentation of the lesion in **(A)**. D.2022.5 Pelvic lymph node under 18F-OC PET, increased distribution of radioactivity is seen, SUVmax=6.2. **(E)** 18F-OC PET image 3 months after 177Lu-DOTA-TATE treatment, the volume of the lesion is significantly reduced compared with that before the treatment, and the level of octreotide expression is significantly reduced compared with the previous one, which is considered to be the residual tumor activity. SUVmax=5.5. **(F)** 18F-OC PET image 3 months after 177Lu-DOTA-TATE treatment, pelvic lymph nodes compared with **(D)**, the expression level of octreotide was significantly reduced, which was consistent with post-treatment changes.

**Figure 3:**
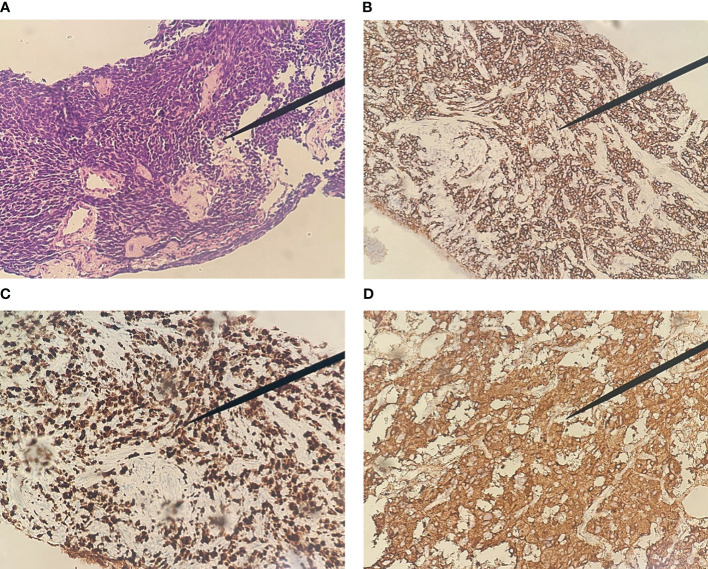
Pathological features of Prostate small cell carcinoma. **(A)** The tumor cells, diminutive in size and arranged in patches or in a nest-like manner, have scant cytoplasm, rendering them nearly nucleated. These nuclei are abundant with chromatin, yet the nucleoli remain indistinct (HE, 200×); Immunohistochemical image of Prostate small cell carcinoma (SP 200×). **(B)** positive immunohistochemical staining for CD56; **(C)** positive immunohistochemical staining of Ki-67(95%); **(D)** positive immunohistochemical staining for Synaptophysin.

A male patient in his 80s presented to the hospital in January 2021 upon the discovery of elevated prostate-specific antigen (PSA) levels during a physical examination. Further investigations at the hospital revealed a PSA level of 33.21 ng/ml. Prostate magnetic resonance imaging (MRI) with T1-weighted, dynamic enhancement, and spectroscopy analysis indicated the presence of prostate tumor with involvement of the prostatic capsule and close association with bilateral seminal vesicles. PSMA PET/CT also support this diagnosis (**Figure 1**). A prostate biopsy was performed, yielding a pathological diagnosis of moderately differentiated adenocarcinoma of the prostate with a Gleason score of 4 + 3 = 7. According to the 2016 WHO/ISUP grading system, the tumor was classified as 85% Grade 4 and 15% Grade 3, categorized as Group III/V. The diagnosis of prostate cancer was confirmed. The patient opted for CAB (bicalutamide + goserelin) therapy, and after three months of treatment, the follow-up PSA measurement showed a level of 0.03 ng/ml, while pelvic MRI indicated a reduction in the size of the prostate cancer lesion. In September, the patient experienced symptoms of urinary frequency, dysuria, and difficulties with urination. A follow-up PSA measurement revealed a level of 0.02 ng/ml; however, due to patient-related reasons, imaging studies were not conducted. In May 2022, the patient presented with symptoms of rectal irritation and underwent further examinations at the hospital. The PSA level indicated 0.01 ng/ml, while the color Doppler ultrasound showed prostate enlargement accompanied by diffuse homogeneous changes, multiple hypoechoic lesions in the prostate, and considering the medical history, suggested treatment-related alterations in the prostate. The residual urine volume in the bladder was approximately 110 ml. Pelvic MRI scans, performed in light of clinical indications for post-treatment evaluation of prostate cancer, revealed involvement of the seminal vesicles, periprostatic tissues, and soft tissues anterior to the rectum. The boundary between the lesion and the posterior wall of the bladder and the anterior wall of the rectum appeared indistinct, implying potential invasion. Enlarged lymph nodes were observed in the lower abdomen, pelvic region, and bilateral iliac vasculature, along with osseous changes in the left ilium and sacrum, suggestive of possible metastasis to the left femur. The patient underwent another prostate biopsy, and the pathological findings revealed poorly differentiated carcinoma with a few sieve-like structures locally, invading the skeletal muscle. In conjunction with immunohistochemical results, it corresponded to small cell carcinoma with a small amount of moderately differentiated adenocarcinoma. The immunohistochemistry results demonstrated broad CK expression, CD56 positivity, Synaptophysin positivity, absence of Chromogranin A, absence of TTF-1, slight CK7 positivity in a few glandular structures, weak PSA positivity in glandular structures, P504S positivity in glandular structures, slight GATA-3 positivity in glandular structures, local basal cell positivity for P63 in glandular structures, local basal cell positivity for 34βE12 in glandular structures, absence of LCA, and a Ki-67 index of 95% (**Figure 3**). In order to better assess the patient’s condition, we conducted PSMA PET/CT imaging, which yielded inconsistent results compared to the MRI findings. Subsequently, the patient underwent 18F-AlF-NOTA-octreotide (18F-OC) PET/CT (**Figure 2**), genetic testing, and other examinations. The 18F-OC scan revealed multiple hypermetabolic lesions within the prostate, indicative of malignant transformation, potentially involving the seminal vesicles, bladder wall, and rectum. Additionally, there were hypermetabolic lymph nodes throughout the body, suggestive of lymph node metastasis. Metabolic lesions with increased activity were observed in the midshaft of the right humerus and the left ilium, indicating possible bone metastasis. Furthermore, a high metabolic soft tissue density lesion was identified in the tail of the pancreas, suggesting the possibility of metastatic lymph nodes. Genetic testing revealed no mutations in KRAS Exon2 (codon 12/13), KRAS Exon3 (codon 61), BRCA1, and BRCA2. Following discussions among the hospital’s experts, the patient and their family chose to undergo 177Lu-DOTA-TATE therapy. In June, the patient received the first round of 177Lu-DOTA-TATE treatment (7.4 GBq, 200 mCi). For renal protection, an intravesical amino acid solution (25 g lysine and 25 g arginine mixed in 1 L of saline) was given concurrently for 4 hours starting 30 minutes before the infusion of the radiopharmaceuticals. Two months after the treatment, symptoms such as rectal irritation, urinary frequency, dysuria, and difficulties with urination gradually subsided. Tests showed no significant impairment of liver or kidney function. There were no significant toxic side effects observed. In August, the patient underwent the second round of treatment. One month after the second treatment, a follow-up 18F-OC scan indicated a noticeable reduction or disappearance of the original lesions in comparison to previous scans, with metabolic activity trending towards normal (**Figure 2**). The patient has been followed up at our hospital since the second treatment, and no disease progression has been observed thus far.

**Figure 4:**
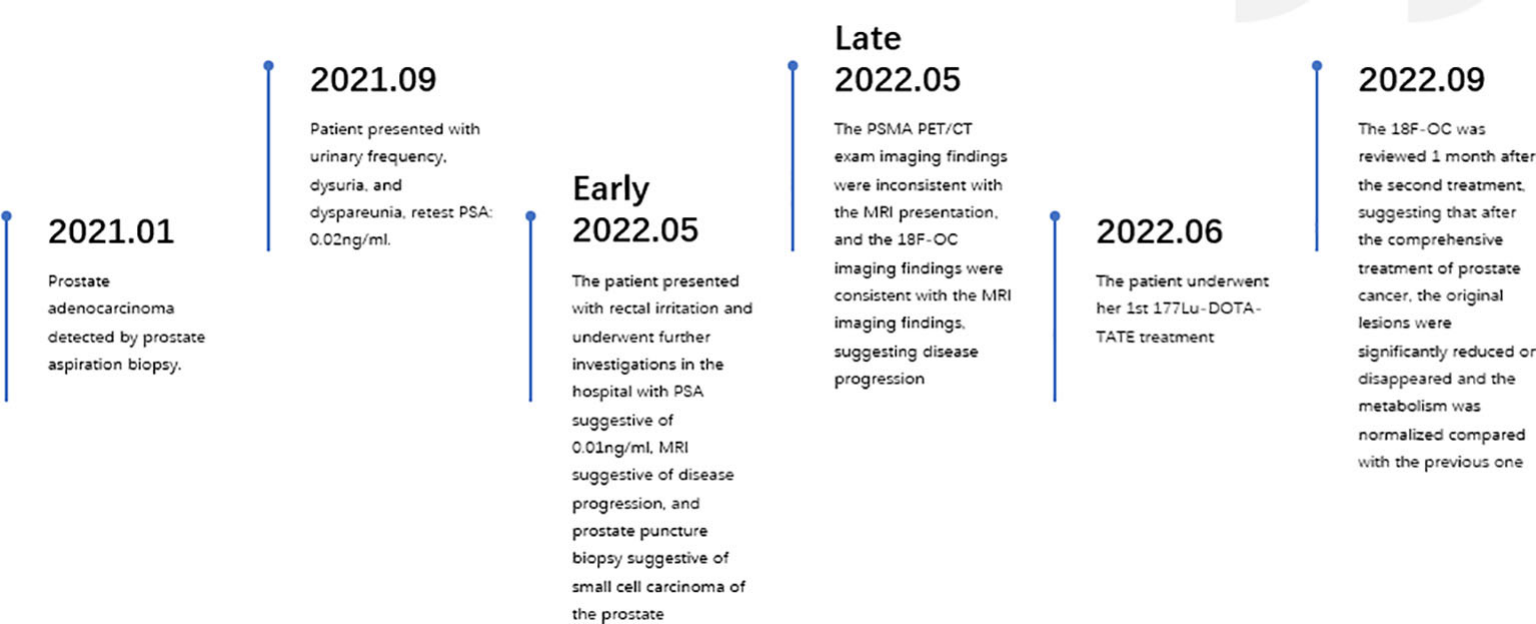
Illustrates a timeline of relevant diagnoses and treatments.

**Figure 4** illustrates a timeline of relevant diagnoses and treatments.

## Discussion

Small cell carcinoma of the prostate (SCCP) is considered a poorly differentiated neuroendocrine cancer with a more rapid disease progression compared to adenocarcinoma. As the disease advances, most patients exhibit rapidly worsening urinary obstruction symptoms, along with accompanying lower back pain and hematuria. Despite its aggressive nature, SCCP often presents with low levels of prostate-specific antigen (PSA), as observed in this case where the PSA level remained within normal range during combined androgen blockade therapy. Currently, conventional imaging methods do not directly differentiate between SCCP and prostate adenocarcinoma. However, with the development of positron emission tomography/computed tomography (PET/CT) technology, various PET tracers can now be used for imaging prostate cancer and neuroendocrine differentiation. Overexpression of somatostatin receptors (SSTR) is one of the key features of neuroendocrine tumors. Combining the advantages of radiolabeling with chelating agents and the imaging and logistical benefits of fluorine-18, we chose to utilize 18F-OC for our purposes (1). Currently, the diagnosis of SCCP primarily relies on histological features and immunohistochemistry. As the disease progresses, prostate cancer exhibits varying degrees of tumor composition changes. This phenomenon may be attributed to the administration of novel androgen receptor-targeted therapies (such as abiraterone and enzalutamide). Adenocarcinoma cells can acquire neuroendocrine carcinoma (NEC) markers while losing androgen receptor (AR) expression, leading to transdifferentiation into SCCP cells (2). However, due to the low incidence rate of this disease, further research is needed to enhance our understanding of this trend. Therefore, we opted to perform a second biopsy on this patient to gather more information. Under microscopic examination, SCCP exhibits similarities to small cell carcinoma, while immunohistochemically, SCCP typically does not express markers commonly found in prostate cells, such as prostate-specific antigen (PSA). Instead, it characteristically expresses neuroendocrine lineage markers such as chromogranin A, synaptophysin, TTF-1, somatostatin receptor (SSTR), and CD56 (3). Additionally, the Ki-67 proliferation index is generally higher, indicating a more aggressive biological behavior of the tumor. In a study, it was found that 75% of SCCP specimens showed strong expression of FOXA2, suggesting that FOXA2 could be a sensitive and specific molecular marker with potential significant value in the pathological diagnosis of small cell neuroendocrine carcinoma (4). Additionally, another study elucidated the exosomal proteome in SCCP cell models through mass spectrometry. This investigation identified thrombospondin-1 (TSP1) as a specific biomarker, suggesting its potential utility as a novel non-invasive tool for identifying SCCP and guiding treatment decisions (5). However, a substantial number of clinical samples are still required to validate the effectiveness of this methodology.

Currently, there is no standardized approach for the treatment of this condition. Radical prostatectomy proves effective in non-metastatic SCCP cases, as it can prolong median survival and achieve complete remission in a minority of patients. However, for the majority of SCCP cases, metastasis has already occurred at the time of diagnosis, resulting in missed opportunities for optimal surgical intervention.

Previous research indicates that the loss of androgen receptor (AR) expression in SCCP patients does not yield benefits from androgen deprivation therapy (ADT) and may even accelerate disease progression. ADT is primarily suitable for mixed-type SCCP patients with elevated prostate-specific antigen (PSA) levels but is not the predominant treatment regimen. Therefore, a comprehensive treatment approach based on chemotherapy is recommended for SCCP management. The guidelines from the National Comprehensive Cancer Network (NCCN) endorse the EP regimen as the standard treatment for SCCP. Commonly employed chemotherapeutic agents include platinum-based drugs, etoposide, docetaxel, paclitaxel, cyclophosphamide, and topotecan. Drawing insights from treatment protocols for small cell lung cancer, the EP regimen (etoposide + cisplatin) is frequently utilized for SCCP treatment. Overall, platinum-based chemotherapy exhibits a certain degree of efficacy, although the duration of response is limited. Further clinical validation is required for alternative chemotherapy regimens.

In the realm of immunotherapy and targeted therapy, Aurora-A kinase inhibitors such as alisertib, as well as PARP inhibitors like olaparib (OLA) and talazoparib (TALA), may potentially yield certain effects in treating SCCP. However, SCCP being a rare tumor, further evidence is needed to substantiate the efficacy of these drugs. Prostate-specific membrane antigen (PSMA), a transmembrane glutamate carboxypeptidase, is highly expressed on prostate cancer cells. 177Lu-PSMA-617 selectively delivers beta particle radiation to PSMA-positive cells and the surrounding microenvironment, while preserving the majority of normal tissues in patients confirmed through imaging for radioactive isotope binding (6). Hence, patients with low PSMA expression or those who are FDG-positive but PSMA-negative (inconsistent FDG-avidity) do not derive benefits from it (7). However, the specific threshold for low PSMA expression has not been established, indicating the need for further research. In this case, the patient has been diagnosed with SCCP and showcases diminished PSMA expression. Therefore, based on the pronounced expression of SSTR in neuroendocrine tumors, we have chosen to administer 177Lu-DOTA-TATE【177Lu-labeled sst analogue, specifically 1,4,7,10-tetraazacyclododecane-NI, NII, NIII, NIIII-tetracetic acid (D) Tyr3-octreotate (DOTATATE), is an established medication used for the treatment of patients with inoperable or metastatic NETs (8)】. Upon reviewing the existing literature, it was found that 177Lu-DOTA-TATE is commonly employed in the management of gastrointestinal neuroendocrine tumors. NETTER-1 represents the pioneering randomized phase III clinical trial investigating PRRT, where a total of 229 patients with intestinal neuroendocrine tumors were randomly assigned into two groups: (1) The treatment group consisted of 116 individuals who received 177Lu-DOTA-TATE therapy. They were administered a dosage of 7.4 GBq every 8 weeks (four intravenous infusions), including long-acting and repeatable octreotide (LAR), supplemented by a muscular injection every 4 weeks. (2) In contrast, the control group included 113 participants who only received octreotide LAR, 60 mg, once every 4 weeks. The study report indicates that compared to high-dose octreotide, 177Lu-DOTA-TATE treatment resulted in a remarkable 79% reduction in the risk of progression or death (p>0.001, HR 0.21) (9). Hence, we embarked on the first attempt to incorporate 177Lu-DOTA-TATE into the treatment regimen for prostate small cell carcinoma (SCCP). 177Lu-DOTA-TATE Common adverse reactions include lymphopenia, vomiting, and nausea. Serious adverse reactions include myelosuppression, nephrotoxicity, and hepatotoxicity (9). In this case, the patient exhibited no significant adverse effects after undergoing two treatment cycles. Notably, the existing lesions diminished in size, and the metastatic foci disappeared. This compelling evidence substantiates the efficacy of 177Lu-DOTA-TATE in SCCP therapy, providing a viable approach for its management. Despite its limited clinical implementation thus far, the potential significance of 177Lu-DOTA-TATE in future SCCP treatments cannot be overstated.

## Conclusion

SCCP is a rare malignant tumor with clinical and radiological manifestations that lack specificity, and the PSA level is not elevated. Consequently, misdiagnosis and missed diagnosis are common in patients with this condition. Opting for 18F-OC is an ideal diagnostic method, and histopathological examination can confirm the diagnosis. Surgical intervention is an effective treatment approach, although postoperative adjuvant chemotherapy yields suboptimal results. Peptide receptor ribonucleic acid therapy (PRRT) has been used among clinical treatments, and it has been suggested that PRRT is very effective in terms of progression-free survival (PFS), symptom control, and quality of life; however, the beneficial effects on outcome survival need further validation (10). 177Lu-DOTA-TATE demonstrates favorable efficacy in treating neuroendocrine tumors, but its current application primarily focuses on gastrointestinal neuroendocrine tumors. In this case, the attempt to employ 177Lu-DOTA-TATE for treatment yielded promising results. Clinical physicians should familiarize themselves with the clinical and pathological features of SCCP, as well as the available treatment modalities, in order to prolong patient survival and enhance cure rates. Moreover, we recommend adopting a multidisciplinary approach, involving urologists, oncologists, pathologists, and radiologists, to collaboratively discuss treatment plans and improve the health and quality of life for patients.

## Data availability statement

The original contributions presented in the study are included in the article/Supplementary Material. Further inquiries can be directed to the corresponding author.

## Ethics statement

Written informed consent was obtained from the individual(s) for the publication of any potentially identifiable images or data included in this article.

## Author contributions

X-YY: Conceptualization, Data curation, Investigation, Writing – original draft, Writing – review & editing. Y-QZ: Investigation, Writing – review & editing, Writing – original draft. Conceptualization, Data curation. XL: Conceptualization, Investigation, Writing – review & editing. RT: Investigation, Writing – review & editing, Conceptualization. J-JC: Investigation, Writing – review & editing. G-QL: Investigation, Writing – review & editing. D-YY: Writing – review & editing, Investigation. X-PZ: Data curation, Supervision, Writing – review & editing. BL: Data curation, Supervision, Writing – review & editing. H-JZ: Data curation, Supervision, Writing – review & editing. XL: Data curation, Investigation, Resources, Supervision, Writing – original draft, Writing – review & editing.
